# New cell line development for antibody-producing Chinese hamster ovary cells using split green fluorescent protein

**DOI:** 10.1186/1472-6750-12-24

**Published:** 2012-05-15

**Authors:** Yeon-Gu Kim, Byoungwoo Park, Jung Oh Ahn, Joon-Ki Jung, Hong Weon Lee, Eun Gyo Lee

**Affiliations:** 1 Process Engineering Center, KRIBB, Daejeon, 305-806, Korea

**Keywords:** Split GFP, FACS, Cell line development, Antibody production, CHO cells

## Abstract

**Background:**

The establishment of high producer is an important issue in Chinese hamster ovary (CHO) cell culture considering increased heterogeneity by the random integration of a transfected foreign gene and the altered position of the integrated gene. Fluorescence-activated cell sorting (FACS)-based cell line development is an efficient strategy for the selection of CHO cells in high therapeutic protein production.

**Results:**

An internal ribosome entry site (IRES) was introduced for using two green fluorescence protein (GFP) fragments as a reporter to both antibody chains, the heavy chain and the light chain. The cells co-transfected with two GFP fragments showed the emission of green fluorescence by the reconstitution of split GFP. The FACS-sorted pool with GFP expression had a higher specific antibody productivity (*q*_Ab_) than that of the unsorted pool. The *q*_Ab_ was highly correlated with the fluorescence intensity with a high correlation coefficient, evidenced from the analysis of median GFP and *q*_Ab_ in individual selected clones.

**Conclusions:**

This study proved that the fragment complementation for split GFP could be an efficient indication for antibody production on the basis of high correlation of *q*_Ab_ with reconstitution of GFP. Taken together, we developed an efficient FACS-based screening method for high antibody-producing CHO cells with the benefits of the split GFP system.

## Background

Chinese hamster ovary (CHO) cells are one of the most widely used host cells for therapeutic protein production. The selection of CHO cells for high therapeutic protein production is of particular interest in CHO cell culture for a wide variety of individual clones for productivity from a random integration and gene amplification system [1]. Due to the large number of candidates that need their productivity evaluated, an efficient high-throughput cell screening system needs to be developed.

Flow cytometry can efficiently evaluate a number of cells at the single-cell level in a short time and isolate a single clone from sub-populations. Various fluorescence-activated cell sorting (FACS)-based cell screening methods have been developed for CHO cells with the benefits of flow cytometry [1]. The use of an antibody conjugated with a fluorescent molecule, which is the target to the membrane-retained therapeutic proteins has been used to find high-producing clones in the combination with FACS. Although its mechanism has not been fully elucidated, the incubation of antibody-producing CHO cells at 4°C could lead to the transient association of the desired antibody with the cell surface [2]. The target antibody at cell surface can be detected by another fluorescent-conjugated antibody and the highly fluorescent-labeled clones are isolated as high producers using FACS system, evidenced from correlating the mean fluorescence intensity with specific productivity (*q*). Similarly, to capture the secreted protein in a small droplet or matrix generated on the cell surface, gel microdrop technology and affinity capture surface display method combined with fluorescent-conjugated antibody have been developed [3,4]. Instead of direct measurement of desired antibody by anchoring membrane, the cell surface reporter, CD20 not normally expressed in CHO cells, is co-expressed with the target protein by internal ribosome entry site (IRES)-linking and the high producer is detected using a fluorescent-conjugated anti-CD20 antibody [5].

Being different from the direct detection of a target protein, intracellular fluorescent materials, such as green fluorescence protein (GFP) and yellow fluorescence protein (YFP), were a widely used fluorescent protein for FACS because of easy activation without co-factors and substrates. Meng et al. (2000) evaluated the usefulness of GFP as an indicator for finding high-producing clone when it is co-expressed with therapeutic protein in one vector system [6]. Due to the close relationship between fluorescence intensity and *q*, clones with high *q* could easily be isolated from sub-populations based on fluorescence level using FACS. In a similar way, clones with high fluorescence level were selected as for high *q* in FITC-conjugated MTX-mediated gene amplification system on the basis of correlation of *q* with amplified gene copy number [7]. However, in the case of isolation of antibody-producing cells, there is a limitation in the applications of these systems because antibody is composed of two fragment proteins which are heavy and light chains. Therefore, Sleiman et al. (2008) developed two color fluorescent protein-based FACS to select clone possessing the high level of the heavy chain and the light chain by detecting GFP and YFP, respectively [8].

For the characteristics of an antibody composed of two fragments, a heavy chain and a light chain, the fragment complementation system can be useful in finding antibody-producing cells. Previously, Bianchi and McGrew (2003) developed an efficient system for the selection of CHO cells with high levels of both antibody chains using a DHFR fragment [9]. The fragment complementation systems for split GFP by means of anti-parallel leucine zipper and EF-hand calcium binding motifs of calbindin D9k have been reported [10-12]. Expression of two fragments of GFP does not achieve folding and fluorescence of GFP by itself. However, GFP is reassembled through the introduction of strong mediator such as anti-parallel leucine zipper and EF-hand calcium binding motifs of calbindin D9k by non-covalent reconnection. Despite the merits in using split GFP, such as the extensive use of flow cytometry and efficient complementation system for GFP, there is, to date, no report introducing it related to cell line development in mammalian cell culture. In the present study, we developed a new cell screening method for high antibody-producing CHO cells based on the reassembly of split GFP combined with FACS.

## Results and discussion

**Figure 1:**
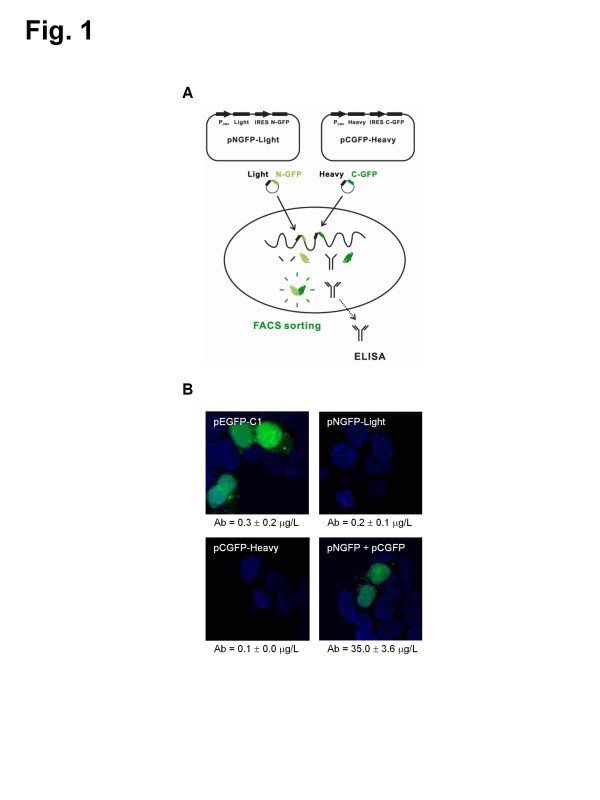
**New cell line development for high antibody-producing mammalian cells.(A)** Schematic diagram for the split GFP-based cell screening method. **(B)** Confocal microscopic images of cells transfected with a GFP-overexpressing vector (pEGFP-C1) or bicistronic vector having GFP fragements and both antibody chains (pNGFP-Light and pCGFP-Heavy). After transfection with these vectors, the cells were cultivated for two days at 30°C. DAPI was used for nucleus staining. Antibody titer was quantified from culture supernatant by ELISA.

To evaluate the role of split GFP as a reporter for antibody production, we constructed an overexpression vector for split GFP and antibody chains expressed simultaneously. Figure 1A shows the schematic diagram representing the way the assembly of split GFP works as a reporter for the selection of antibody-producing cells. The GFP fragments, called N-GFP and C-GFP (described in the ‘Methods’ section), are co-linked with a light chain and heavy chain gene using an IRES sequence, respectively. These constructs containing an IRES sequence, pNGFP-Light and pCGFP-Heavy, led us to hypothesis that the transcription level of the light and heavy chains is highly correlated with that of N-GFP and C-GFP. From this hypothesis, our speculation extends to the idea that the high GFP-expressing clone may be the high antibody-producing clone.

To confirm both the antibody expression and the assembly of the GFP with a constructed vector, a bicistronic vector containing GFP fragments and an antibody gene, pNGFP-Light and pCGFP-Heavy, was transfected into HEK293T cells. As a control, a GFP-overexpressing vector, pEGFP-C1, was also transfected into HEK293T cells. Figure 1B shows the confocal microscopic images of transfected cells and antibody titer produced from them. The emission of green fluorescence from reconstituted split GFP was efficiently detected when the two fragments were co-expressed. In addition, the cells with green fluorescence could produce the antibody simultaneously. The same results were confirmed in CHO-K1 cells (data not shown). Accordingly, we proceeded with these constructs to evaluate the efficiency of this screening system in CHO cells.

**Figure 2:**
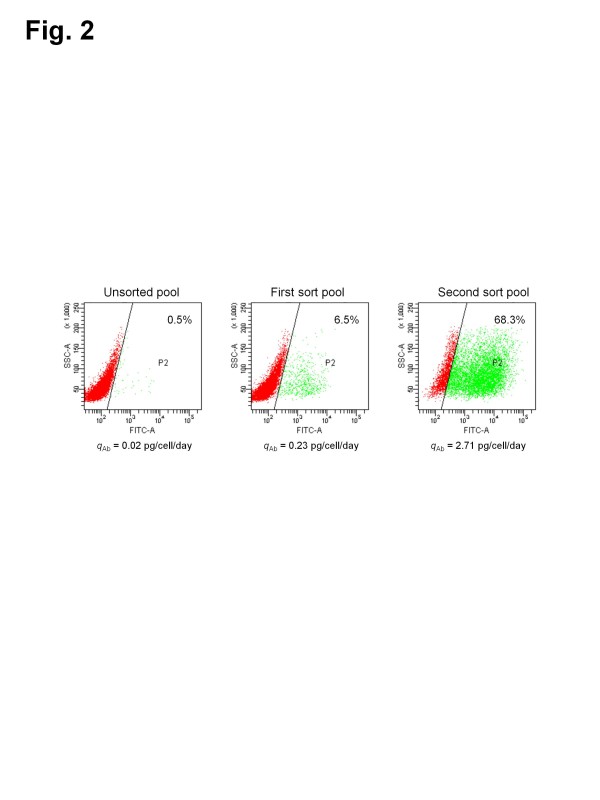
**Flow cytometry analysis for green fluorescence and*q***_**Ab**_**of unsorted cells and FACS-sorted cells having reconstituted GFP.** Dot plots of SSC versus log-FITC fluorescence are shown. Each dot represents a single cell; 10,000 cells are represented in each plot. The line in the plots depicts the cutoff between GFP-positive and GFP-negative cells.

To investigate whether the sorted cells by FACS are the antibody-producing CHO cells, the CHO cells with intracellular green fluorescence (First sort pool and Second sort pool) were generated by two rounds of FACS from the CHO cells co-transfected with pNGFP-Light and pCGFP-Heavy (Unsorted pool). Figure 2 shows the flow cytometry analysis and specific antibody productivity (*q*_Ab_) of unsorted and FACS-sorted cells. The discrimination of cells with green fluorescence was performed on a FITC/SSC diagram. The percentage of the cell population with GFP expression in the First sort pool and Second sort pool was much higher than that in the Unsorted pool. Interestingly, the increase in *q*_Ab_ was highly associated with the increase in the cell population with GFP expression. Taken together, we found that the FACS-sorted cells with GFP expression are the antibody-producing CHO cells.

To evaluate the efficiency of split GFP-based clone selection for high antibody-producing cells, individual clones were isolated from the Unsorted pool and the Second sort pool by the limiting dilution method. Table 1 shows the distribution of 116 isolated clones from the Unsorted pool and the Second sort pool with regard to antibody titer, respectively. All of the Unsorted pool-derived clones have low antibody production (less than 1 mg/L). Eighty-two out of 116 clones in the Second sorted pool have antibody production exceeding 1 mg/L. Moreover, there are four clones having over 10 mg/L in the Second sort pool-derived clones, while there are none in the Unsorted pool-derived clones. Altogether, the clone selection with split GFP-based FACS appears to be an efficient strategy to select high antibody-producing CHO cells.

**Table 1:** Distribution of isolated clones from the Unsorted pool and the Second sort pool for antibody titer

**Criterion (mg/L)**^**a**^	**Unsorted pool**	**Second sort pool**
< 0.5	115/116	33/116
0.5 – 1	1/116	1/116
1 – 5	0/116	28/116
5 – 10	0/116	50/116
> 10	0/116	4/116

^a^Cells were inoculated at a concentration of 3 × 10^5^ cells/mL into a 6-well plate. The samples were measured by ELSIA after 3-days cultivation.

A major issue in the development of a cell screening method using a reporter is to verify the co-relationship between the expression level of the reporter and the antibody. Especially, the screening system dealing with intracellular reporter should be evaluated because antibody is generally secreted protein. In the same context, previous reports utilizing a fluorescent protein as an intracellular reporter proved the mutual relation between productivity of desired protein and fluorescence intensity [6,8].

**Figure 3:**
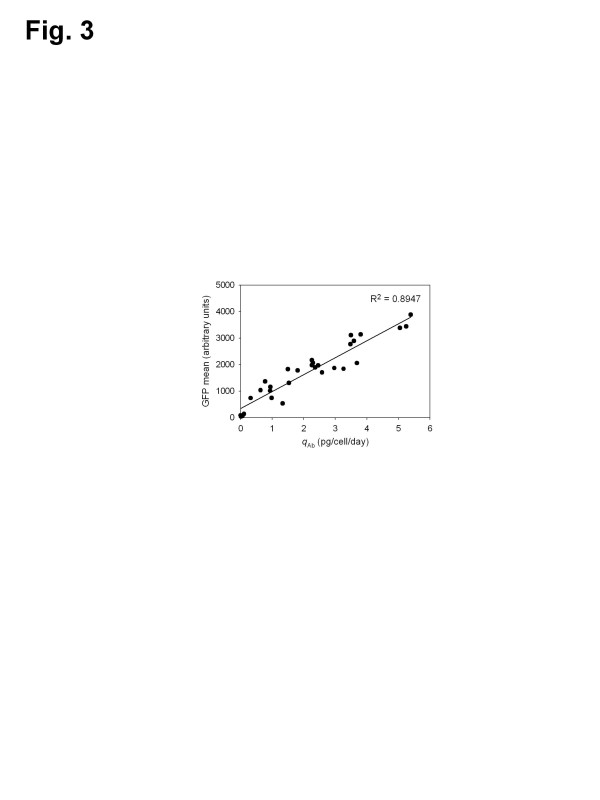
**Relationship between the*q***_**Ab**_**and green fluorescence intensity in 30 selected clones.** Median green fluorescence intensity (GFP mean) and *q*_Ab_ were measured by flow cytometry analysis and ELISA, respectively. The *q*_Ab_ and GFP mean (X, Y) pairs for each clone were plotted to calculate the correlation coefficient (R^2^).

To address this issue, the median GFP and *q*_Ab_ of 30 selected clones were estimated. Figure 3 shows the relationship of the median GFP and *q*_Ab_ for individual selected clones. The *q*_Ab_ is highly correlated with the fluorescence intensity as indicated by the high correlation coefficient (R^2^ = 0.8947). Accordingly, we confirmed that the selected clone having high fluorescence intensity by GFP-based cell sorting is the high antibody producer with high *q*_Ab_.

Another issue that arises with the usage of intracellular reporter is the determination of the optimum time-point for sorting and metabolic burden by co-expressing reporter gene. Unlike the use of non-split fluorescent protein, we can exclude the issue of determining the optimal point in split fluorescent protein because the split fluorescent protein can be assembled at specific conditions, such as low temperature in the case of split GFP. Previously, Bochkov and Palmenberg (2006) showed that the level of gene expression linked with attenuated IRES was about 11-fold lower than wild-type IRES [13]. Also, the modified form of GFP with increased fluorescence intensity was reported [14]. The introduction of attenuated IRES and improved form of GFP can be a solution to reduce the metabolic burden of fluorescent protein.

The intracellular fluorescent protein-based FACS for clone selection in rCHO cells is a reasonable strategy as it does not require complex preparation steps to capture and detect a secreted antibody. Previously, Sleiman et al. (2008) developed an efficient dual intracellular auto-fluorescent protein-associated FACS to select antibody-producing CHO cells with a high level of both antibody chains [8]. In this system, two reporters, GFP and YFP, are applied for the heavy and light chain, respectively. In general, a correct compensation is necessary for accurate analysis when more than two colors are used as a parameter in flow cytometry [15]. Moreover, a similar level of fluorescence intensity in GFP and YFP does not guarantee a similar expression level of the heavy chain and the light chain because fluorescence intensity is a relative value, not an absolute value. Also, the split GFP-based cell screening method developed in this study is advantageous in that one reporter is introduced for quantitative analysis. By employing this type of complementation system, we can omit the process of compensation.

The additional validation for dynamic range of the GFP assay and long-term stability of constructed cell line may be needed to successfully apply the split GFP-based cell screening method for commercial production cell line. The expanded result for dynamic range of GFP expression in high antibody-producing clones with enhanced *q*_Ab_ via gene amplification system and/or *cis*-acting element for augmenting gene expression may be helpful to meet the industrial standard. The genetic instability of antibody-producing CHO cell line during long-term culture in the absence of selective pressure is one of main issues in CHO cell culture [16]. The data provided from long-term culture with constructed cell lines based on split GFP system to determine the association of GFP expression with reduced *q*_Ab_ will be an interesting extension of this work.

## Conclusions

The considerable clonal variation attributed from transfection and gene amplification makes it difficult to establish high-producing rCHO cell line. For large number of analytes, it is necessary to develop an efficient high-throughput cell screening system. In this study, we developed an efficient screening method based on reconstitution of split GFP to select high antibody-producing CHO cells using a FACS analysis. On the basis of correlation between *q* and fluorescence intensity by reconstituting GFP, the fragment complementation system for split GFP could be a powerful tool for antibody production in CHO cells.

## Methods

### Plasmid construction

A modified pIRES vector (BD Biosciences Clontech) with wild-type IRES was used for the construction of pNGFP-Light and pCGFP-Heavy. The light and heavy chain genes were kindly provided by Dr. J. S. Yoo, Pharmabcine Co., Ltd. They were inserted into MCS-A of the pIRES vector to yield pIRES-Light and pIRES-Heavy, respectively. The GFP coding sequence was obtained from pIRES2-EGFP (BD Biosciences Clontech) by PCR amplification. DNA construct for Calbindin D9k including EF1 and EF2 were synthesized in Bioneer Co., Ltd. as described previously [10]. N-GFP encoding N-terminal GFP (amino acid residue 1–158) linked with EF1 via GGSGSGSS and C-GFP encoding C-terminal GFP (amino acid residue 159–239) linked with EF2 via TSGGSG were made by a sequential PCR method as described previously [12,17]. Then, they were inserted into MCS-B of the pIRES vector resulting in pNGFP-Light and pCGFP-Heavy, respectively.

### Cell line and culture maintenance

The CHO-K1 cells (ATCC CRL-61) were plated onto 25 cm^2^ T-flasks (Nunc), and attached for 24 h before transfection. The CHO-K1 cells (1 × 10^6^ cells) were co-transfected with pNGFP-Light (4 μg) and/or pCGFP-Heavy (4 μg) using Lipofectamine^TM^ 2000 (Invitrogen) according to the manufacturer’s protocol. Drug selection was carried out for three weeks by seeding 1 × 10^4^ cells/mL in 75 cm^2^ T-flasks (Nunc) containing Roswell Park Memorial Institute (RPMI; Invitrogen) supplemented with 10% fetal bovine serum (FBS; Invitrogen) and 500 μg/mL G418 (Invitrogen). After drug selection, a stable pool, called the Unsorted pool, was maintained for three days at a concentration of 1 × 10^5^ cells/mL. The Unsorted pool was cultivated for two days at 30°C and then the top 1% of the fluorescence intensity in the Unsorted pool using FACS analysis (See below) was collected and called the First sort pool. The Second sort pool was prepared from the top 1% of the fluorescence intensity of the First sort pool using the same procedure.

### Batch culture

Exponentially growing cells were inoculated at a concentration of 0.7 × 10^5^ cells/mL into 25 cm^2^ T-flasks (Nunc) containing 5 mL of RPMI supplemented with 10% FBS and 500 μg/mL G418, and the plates were incubated in a humidified 5% CO_2_/air mixture at 37°C. Periodically, the 25 cm^2^ T-flasks were sacrificed for determining the viable cell concentration. The cell concentration and viability were estimated with a Cytolecon automated cell imaging counter (CYT-100; ECI Inc.) using the trypan blue dye exclusion method. Culture supernatants, after centrifugation, were aliquoted and kept frozen at −70°C for later analyses.

### Enzyme-linked immunosorbent assay (ELISA)

The secreted antibody concentration was quantified by an enzyme linked immunosorbent assay (ELISA). Briefly, 96-well plates (Nunc) were coated with anti-human IgG (Sigma) and blocked with 2% bovine serum albumin (BSA; Sigma) in phosphate buffered saline (PBS) containing 0.1% Tween-20 (Sigma). Human IgG standard (Sigma) and culture supernatants diluted with a blocking buffer were loaded into each well and treated with peroxidase conjugated goat anti-human IgG (Sigma) in the blocking buffer. 3,3',5,5'-tetramethyl benzidine (TMB; Sigma) was used as a substrate and 1 M H_2_SO_4_ was used to stop the reaction. Absorbance was measured by an UV ELISA reader (Bio-Rad) at 450 nm.

### Fluorescence-activated cell sorting (FACS) analysis

The cells were cultivated for two days at 30°C and then were resuspended with PBS. The FACSAria™ system (BD Biosciences Clontech) was used to estimate the green fluorescence intensity of various clones as described previously [18]. Green fluorescence at 525 nm was detected through an FL1 set at a PMT voltage of 400, with a logarithmic gain. Ten thousand cells were analyzed for each sample.

### Fluorescence microscopy

The HEK293T cells (ATCC CRL-11268) were plated onto RS-treated Lab-Tek®II Chamber Slides (Nunc), and attached for 24 h before transfection. For transient expression, HEK293T cells (1 × 10^5^ cells) were co-transfected with pNGFP-Light (0.4 μg) and/or pCGFP-Heavy (0.4 μg) using Lipofectamine^TM^ 2000 (Invitrogen) according to the manufacturer’s protocol [19]. Transfected cells were cultivated for two days at 30°C and then were fixed in 3.7% formaldehyde in PBS for 20 min. The chambers were removed and the slides were sealed with cover slips after adding a Vectashield mounting medium (Vector Laboratories) with DAPI for nucleus staining. All images were collected by an LSM 510 confocal laser-scanning microscope (Zeiss) and processed using the Adobe Photoshop software.

### Evaluation of specific antibody productivity (*q*_Ab_)

The specific antibody productivity (*q*_Ab_) was evaluated as described previously [20]. The *q*_Ab_ was calculated from the plot of antibody concentration versus the time integral of viable cells during day 2 and day 4.

## **Competing interests**

The authors declare that they have no competing interests.

## Authors’ contributions

YGK, JKJ, HWL, and EGL designed the research. YGK, BP, and JOA performed all the experiments and analyzed the data. YGK prepared the initial draft of the manuscript. EGL conceived of the study, coordinated all the components of the project, and prepared the final manuscript. All authors have read and approved the final manuscript.
